# High-salt diet decreases mechanical thresholds in mice that is mediated by a CCR2-dependent mechanism

**DOI:** 10.1186/s12974-020-01858-6

**Published:** 2020-06-09

**Authors:** Anni Fan, Oladayo Oladiran, Xiang Qun Shi, Ji Zhang

**Affiliations:** 1 The Alan Edwards Centre for Research on Pain, McGill University, 740, Dr. Penfield Avenue, Montreal, QC, H3A 0G1 Canada; 2 Department of Microbiology and Immunology, Faculty of Medicine, McGill University, Montreal, Canada; 3 Department of Neurology and Neurosurgery, Faculty of Medicine, McGill University, Montreal, Canada; 4 Faculty of Dentistry, McGill University, Montreal, Canada

**Keywords:** High-salt diet, Pain sensitivity, Monocytes, Macrophages, Microglia, CCR2, Inflammation

## Abstract

**Background:**

Though it is well-known that a high-salt diet (HSD) is associated with many chronic diseases, the effects of long-term high-salt intake on physiological functions and homeostasis remain elusive. In this study, we investigated whether and how an HSD affects mouse nociceptive thresholds, and myeloid cell trafficking and activation.

**Methods:**

Healthy C57BL/6 male and female mice were fed an HSD (containing 4% NaCl in chow and 1% NaCl in water) from the time of weaning for 3 to 4 months. Circulating monocytes, nerve macrophages, spinal microglia, and associated inflammatory responses were scrutinized using flow cytometry, immunohistochemistry, and quantitative real-time polymerase chain reaction (qPCR) approaches. Mouse pain sensitivity to mechanical stimuli was monitored with von Frey tests along the experimental duration.

**Results:**

Mice on an HSD have reduced mechanical thresholds. They feel more pain than those on a normal diet (ND), e.g., regular laboratory chow (0.3% NaCl in chow). An HSD induced not only a remarkable expansion of circulating monocytes, CCR2^+^Ly6C^hi^ inflammatory monocytes in particular, but also an accumulation of CD11b^+^F4/80^+^ macrophages in the peripheral nerves and an activation of Iba-1^+^ spinal microglia. Replacing an HSD with a ND was unable to reverse the HSD-induced mechanical hypersensitivity or rescue the altered immune responses. However, treating HSD-fed mice with a chemokine receptor CCR2 antagonist effectively normalized the pain thresholds and immune cell profile in the periphery and spinal cord. An HSD failed to alter pain thresholds and myeloid cell activation in CCR2-deficient mice. Spinal microglial activation is required for HSD-induced mechanical hypersensitivity in male, but not in female mice.

**Conclusion:**

Overall, this study provides evidence that an HSD has a long-term impact on physiological function. CCR2-mediated cellular response, including myeloid cell trafficking and associated inflammation, plays pivotal roles in salt-dietary modulation of pain sensitivity.

## Background

A healthy diet provides human bodies with essential nutrients and certain “signals” needed to maintain wellness. Sodium plays a vital role in the regulation of many physiological functions, including blood volume maintenance, water balance, cell membrane potential, acid-base balance, and nerve conduction. While the minimum physiological requirement for sodium is 500 mg per day and the dietary guidelines for Americans [1] suggests less than 2300 mg of daily sodium intake, adult men in the USA consume on average 4240 mg per day while women consume 2980 mg, primarily from processed food.

Excess salt intake has been linked to many chronic diseases, including hypertension, cardiovascular disease, kidney dysfunction, and osteoporosis [2–5]. While an increase in salt-enriched “fast food” consumption has been considered a putative factor for many pathological conditions, there is no (animal or human) data available to delineate whether a long-term high-salt diet (HSD) may disturb pain sensitivity. The ability to sense pain is one of the prerequisites for survival. By detecting harmful stimuli and responding quickly, people can protect themselves from damage. Thresholds to painful stimuli may vary among individuals, often attributed to genetic variances, but it is interesting to examine whether a subject’s environment, lifestyle, or diet impacts their ability to sense pain.

While noxious signals are detected, transmitted, and perceived within the sensory nervous system, from the periphery to the central, recent evidence strongly suggests that the immune system, once activated, may be involved in modulating neuronal function, which may result in altered pain behavior [6, 7]. Several studies have revealed that an HSD affects the immune system, including both innate and adaptive immunity. High salt levels increase the expression of proinflammatory molecules while decreasing the anti-inflammatory ones in both human and mouse macrophages. This leads to a salt-specific macrophage activation [8]. High salt levels also alter the phenotypes of T cells by inducing pathogenic Th17 development [9] and inhibiting the suppressive function of FoxP3^+^ T regulatory (Tregs) [10, 11]. Experimental autoimmune encephalomyelitis (EAE) was exacerbated in mice on an HSD [12]. In addition, excess salt aggravated cerebral blood-brain barrier disruption in mouse models of cerebral ischemia, which facilitated leukocyte trafficking [13]. Excessive levels of salt likely have pro-inflammatory properties. Nevertheless, whether and how HSD-associated inflammation affects pain sensitivity remains to be determined.

In this study, we aim to 1) determine whether dietary habits can impact an individual’s pain threshold by assessing the ability of mice on a long-term HSD to sense pain and 2) investigate how an HSD disturbs the homeostasis of the immune and nervous systems, leading to altered pain sensitivity. We observed that mice on a long-term HSD felt more pain. The HSDs triggered myeloid cell activation, including an expansion of circulating monocytes, an increased recruitment of nerve macrophages, and spinal microglia. Innate immune cells were polarized toward proinflammatory status by continued high-salt feeding. Once established, an HSD-induced altered immune function and mechanical hypersensitivity were irreversible even if the subject returned to a normal diet (ND). Chemokine receptor CCR2-mediated myeloid cell trafficking/activation played a crucial role in this cascade. Spinal microglia activation is required for HSD-induced hypersensitivity in male but not in female mice.

## Materials and methods

### Animals

C57BL/6 and CCR2 knock out (CCR2KO) male and female mice were bred in house. There were three to five mice per cage in a temperature and humidity-controlled room that maintained a 12/12-h light/dark cycle. Mice had free access to water and food ad libitum. Animal body weight was monitored weekly during dietary intervention. Behavioral experiments were conducted between 9:00 AM and 3:00 PM. All experiments were approved by the Institutional Animal Care and Use Committee of McGill University (Permit # 5775) and conformed to the ethical guidelines of the International Association of the Study of Pain.

### Animal feeding paradigm

The HSDs were initiated during the weaning period (3 weeks old). The HSD regimen consisted of 4% NaCl in chow and 1% NaCl in water [12, 14]. Mice on a normal diet (ND) received regular laboratory chow [0.3% NaCl in the pelleted food from the Teklad irradiated laboratory animal diet (Envigo)] and distilled water. According to the experimental design, the HSD was maintained for a duration of 1.5 to 4 months. C57BL6 mice were resistant to HSD-induced hypertension [15] and their sodium plasma concentration was not affected following 3 months HSD feeding (data not shown). In most measurements, we did not observe significant differences between HSD-fed male and female mice. Therefore, unless otherwise specified, results represent the combined data collected from both male and female mice.

### Pain behavioral tests

Pain behavioral monitoring began after 3 weeks of HSD/ND feeding. Mice were habituated to the testing environment for at least 2 days before testing. The investigator responsible for the behavioral test was blinded to the treatment conditions. The von Frey Test was used to assess paw sensitivity to mechanical stimuli. Mice were placed in a plastic cage with a wire mesh floor that allowed access to the paws. Calibrated monofilaments (Stoelting) were applied perpendicular to the plantar surface of the hind paw using the up-down method. The paw was touched using a set of eight calibrated von Frey hairs with ranging intensity (from 0.008 to 1.40 g of force). A sharp withdrawing of the paw and immediate flinching upon the removal of the monofilaments were considered a positive response. Two consecutive tests, separated by at least 1 h, were performed, and the average result of the two tests was recorded as the 50% paw withdrawal thresholds.

### Flow cytometry

Single cell suspension from blood, sciatic nerve, and spinal cord was prepared as previously described [16]. In short, whole blood was collected from the sub-mandibular vein plexus of mice and kept in in Alsever’s solution (Gibco) to prevent coagulation. After a brief spin down and the removal of the supernatant, samples were incubated in ACK lysing buffer (Thermo Fisher Scientific) at room temperature for 5 min to deplete erythrocytes. Sciatic nerves (bilateral, 2.2 cm each) and lumbar spinal cord (1 cm) were obtained after being perfused transcardially with 0.9% saline. Tissue samples were diced into small pieces (approximately 1 mm^3^) and incubated in RPMI-1640 medium containing collagenase (1.6 mg/ml, Sigma-Aldrich) and DNAase (250 units/ml, Sigma-Aldrich) for digestion at 37 °C for 30 min. The digested samples were filtered through a 70-μm cell strainer to remove debris. After filtration and washing, single cells were incubated with a 2.4G2 blocking buffer for 30 min at 4 °C and then stained with specific fluorochrome-conjugated Abs for 30 min at 4 °C. Viability dye (Fixable Viability dye eFluor780, 1:50, eBioscience) was included in the staining. Counting beads (eBioscience) were also added to each sample to quantify the number of cells per microliter of blood, per nerve, and per 1 cm spinal cord. For intracellular staining, samples were blocked and labeled first with Abs for surface markers, and then fixed and permeabilized using BD cytofix/cytoperm TM plus kit. This was followed by incubation with Abs against intracellular proteins (CD206, Ki67). Data was acquired with FACS Canto II (BD) and analyzed with Flowjo software. Detailed information regarding the Abs used here is listed in Table 1.

**Table 1:** List of antibodies used for cytoflowmetry analysis

Antibodies	Dilution	Source	Catalog number	Clone
CD45	1:50	BD pharmingen	560501	30-F11
CD11b	1:50	BD pharmingen	552850	M1/70
CD115	1:50	eBioscience	55-1152-82	AFS98
CD86	1:50	eBioscience	12-0862-82	GL1
CD206	1:100	AbD Serotec	MCA2235A6477	MR503
CCR2	1:50	Biolegend	150604	SA203G11
CX3CR1	1:50	Biolegend	149005	SA011F11
F4/80	1:50	eBioscience	45-4801-82	BM8
Ki67	1:50	eBioscience	12-5698-82	SOIA15

### Immunohistochemistry

Mice were transcardially perfused with 0.9% NaCl, which was then followed by 4% paraformaldehyde (PFA) (in 0.1 M sodium phosphate buffer with 7.4 pH) perfusion. Spinal cords and sciatic nerves were harvested for immunohistochemistry staining. Tissues were post-fixed overnight in 4% PFA at 4 °C and then transferred to a 30% sucrose in phosphate buffer until cryostat/microtome sectioning. Sciatic nerves (14 μm) and lumbar spinal cord sections (25 μm, free-floating) were first incubated in a blocking buffer (3% normal rabbit serum, 1% bovine serum albumin, and 0.25% Triton X-100 in 1X Tris-buffered saline) for 1 h at room temperature then incubated with the respective antibodies (Table 2) overnight at 4 °C. This was followed by a 1-h incubation with fluorochrome-conjugated secondary antibodies and DAPI for nuclear staining. After staining, Vectashield mounting medium (Vector Laboratories) was applied for fluorescent microscopic examination.

**Table 2:** List of antibodies used for cytoflowmetry analysis

Antibodies	Dilution	Source	Catalog number
F4/80	1:1000,	Serotec	MCA497GA
Iba-1	1:1000	Wako	019-19741
C16/CD32	1:200	R&D	AF1460

### Image analysis and quantification

Sciatic nerve and lumbar spinal cord sections were examined under the fluorescent Olympus BX51 microscope bearing a color digital camera (Olympus DP71). Images were acquired under the same set of parameters (image processing, picture size, and orientation) and at the optimal intensity to avoid an over-saturation of signals. Ionized calcium binding adaptor molecule 1 (Iba-1) and CD16-32 stained cells were quantified using the same area of interest (AOI) on the superficial layers of the spinal dorsal horns for all samples. Eight to ten sections per animal and three to four animals per group were included for quantification using ImagePro software. The investigator was blinded to the treatment conditions.

### Plasma and urine osmolality measurement

Male and female mice plasma and urine osmolality (mOsm/kg) were measured using the freezing point osmometer. The plasma/urine samples (20 μl) were collected from mice under ND and 1 or 3 months of HSD feeding. The final values of plasma/urine osmolality were reported as the average of triplicate measurements.

### RNA extraction and real-time quantitative PCR

Whole blood was collected from the sub-mandibular vein plexus and lyzed by Ack Lysing Buffer. Sciatic nerves were harvested following transcardiac perfused with 0.9% NaCl fresh-frozen at − 80 °C. Tissue samples were homogenized in a Precellys 24 tissue homogenizer (Bertin Technologies) using two 20-s pulses at 5600 rpm. The total RNA of the blood and sciatic nerves was extracted using TRIzol Reagent (Ambion Life Technologies, Carlsbad, CA) according to the manufacturer’s instructions. The ratio of absorbance at 260 and 280 nm was measured by a Nanodrop2000 (Thermo Scientific, Wilmington, DE) to assess purity and the concentration of RNA. For reverse transcription, 2.5 μg template RNA was added to SuperScript IV Reverse Transcriptase (Invitrogen). After reverse transcription, quantitative RT-PCR was run using 2.5 μl cDNA and SYBR green chemistry (Bio-Rad) following the supplier’s protocol and a cycling program of 2 min at 95 °C, followed by 40 cycles of 95 °C for 3 s and 60 °C for 30 s, 15 h at 70 °C, and hold at 4 °C on the Rotor-Gene Q (QIAGEN). Mouse qPCR primer sequences for cytokines tested are listed in Table 3. Data was analyzed with the ΔΔCt method using GAPDH as a housekeeping gene [17].

**Table 3:** Primer sequences used for real-time quantitative PCR experiments

	Forward	Reverse
GAPDH	5′ AAT GCA TCC TGC ACC ACC AAC T-3′	5′ AGT GAT GGC ATG GAC TGT CGT CAT-3′
IL-1β	5′ CTA TAC CTG TCC TGT GTA-3′	5′ GCT CTT GAC TTC TAT CTT GTT G-3′
ΙL-6	5′ CTG AA CTT CCA GAG ATA C-3′	5′ TTC ATG TAC TCC AGG TAG-3′
CCL2	5′ CTA CTC ATT CAC CAG CAA GA-3′	5′ TCA GCA CAG ACC TCT CTC-3′
CCR2	5′ AGA AGA GG CAT TGG ATT-3′	5′ CGT GGA TGA ACT GAG GTA-3′

### CCR2 antagonist and minocycline treatment

RS 102895 hydrochloride (Sigma-Aldrich), a chemokine receptor CCR2 antagonist [18], was given either intraperitoneally (I.P.) or intrathecally (I.T.) to male/female mice after 3 months of HSD feeding. RS 102895 was administered daily at a dosage of 15 mg/kg of body weight, I.P. or I.T, for two consecutive days before tissue collection. Minocycline was given I.P. to a separate group of HSD-fed mice, 25 mg/kg, for three consecutive days. Behavioral tests were performed before the injection (as baseline value) and 24 h after each drug administration.

### Statistical analysis

All data is presented as mean ± SEM. GraphPad Prism (v. 7.0) was used for statistical analysis. The different statistical analyses used in each experiment are indicated in the figure legends. In general, an unpaired Student’s *t* test was used for single comparisons between groups, and a two-way ANOVA, followed by Bonferroni’s post hoc analysis tests, was used for multiple comparisons. For all analyses, statistical significance was assigned at *p* < 0.05.

## Results

### An HSD induces long-lasting mechanical hypersensitivity in mice, which is not made reversible by returning to a normal diet

**Fig. 1:**
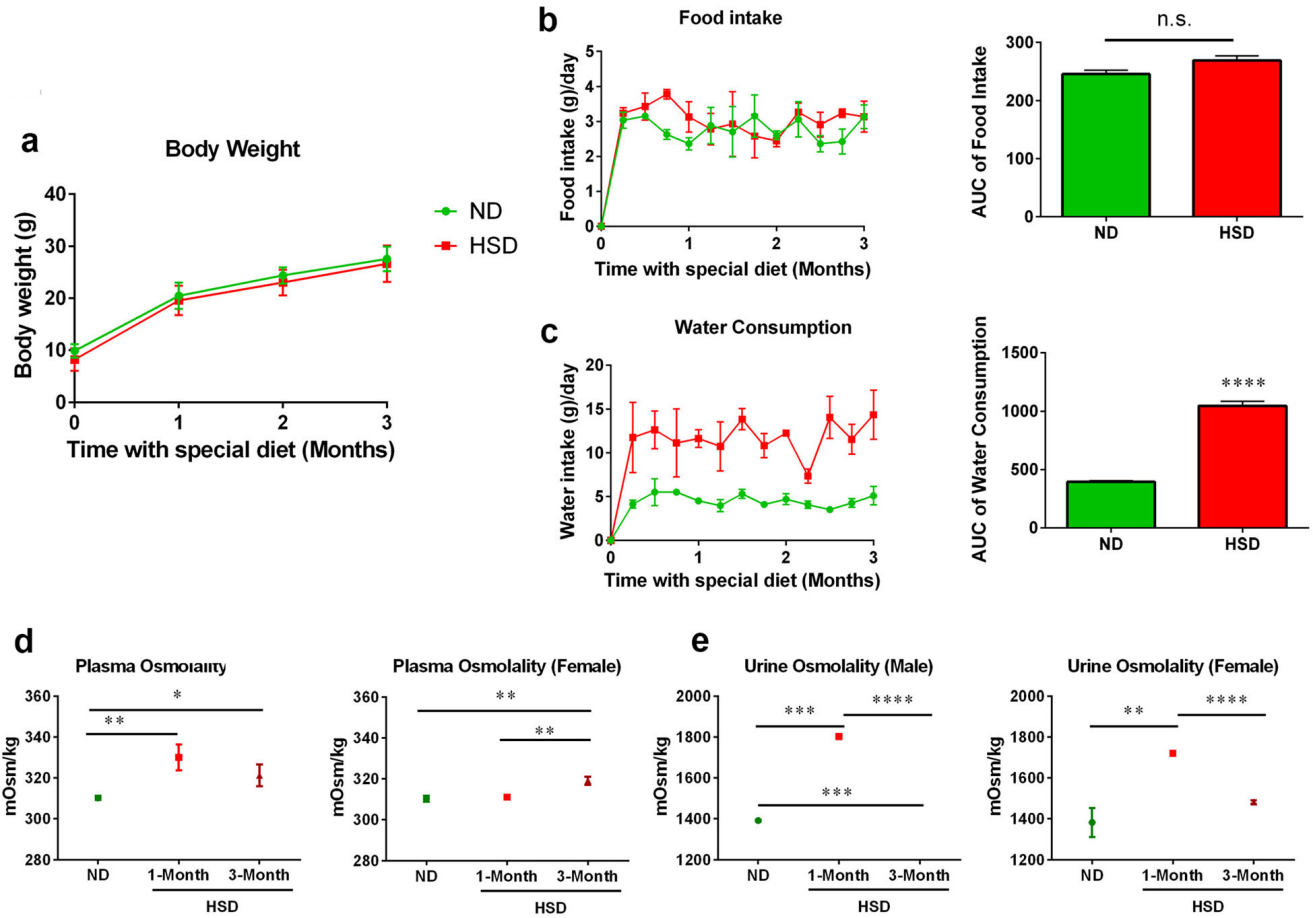
An HSD has no effect on body weight and food intake but does increase water consumption, which affects mouse plasma and urine osmolality. **a** The bodyweights (g) of male/female mice on HSD and ND were recorded for 3 months, *n* = 10–14/group. No significant difference was observed between HSD and ND fed mice. **b** High-salt and regular laboratory chow intake (g) was measured, *n* = 10–12/group. No significant difference was observed between HSD and ND fed mice. **c** Average distilled water and 1% NaCl water (g) consumption were recorded on a weekly basis, *n* = 10–12/group. Mice on an HSD drunk significantly more water than mice on a ND. Male and female mice plasma (**d**) and urine (**e**) osmolality (mOsm/kg) was monitored at 1 month and 3 months during special diet feeding, *n* = 3–4/group. All data were presented as mean ± SEM, and data was analyzed with unpaired *t* test, *p < 0.05, **p < 0.01, ***p < 0.001, ****p < 0.0001

To mimic the circumstances in certain regions of the world wherein people grow up with a higher-than-average salt intake [19], we began feeding mice an HSD directly after weaning, at the age of 3 weeks. There was no difference in the amount of body weight gained between HSD and ND fed mice (Fig. 1a). Mice in both groups experienced a rapid increase in body weight in the first month and afterwards gained weight at a steady rate (Fig. 1a). In addition, mice did not appear to be obsessed with high-salt food because their daily food intake was similar in the HSD and ND groups (Fig. 1b). However, mice on an HSD had a significant increase in water consumption (Fig. 1c). We also measured plasma and urine osmolality to understand the impact of long-term HSD on osmoregulation. Three months of high-salt feeding increased plasma osmolality in both male and female mice, while 1 month of an HSD only elevated plasma osmolality in male mice but not female mice. This indicates that male mice have a quicker response to excessive salt than females (Fig. 1d). Interestingly, for both male and female mice, 1 month of excessive salt intake initially induced an increase in urine osmolality, which was normalized after 3 months of an HSD (Fig. 1e).

**Fig. 2:**
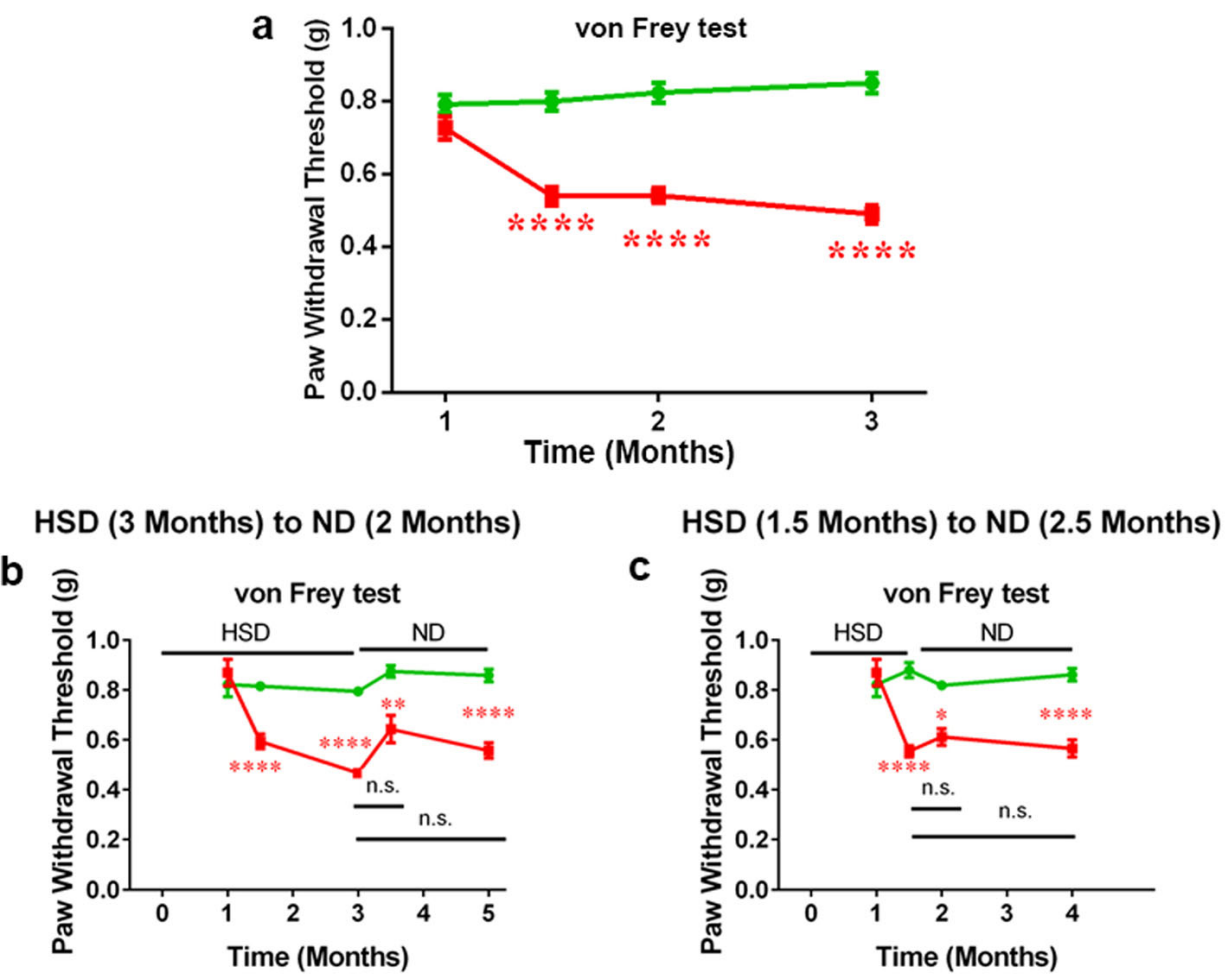
An HSD induces long-lasting hypersensitivity in mice, which is not reversible by returning to a ND. **a P**aw withdrawal thresholds were assessed using the von Frey test. Mechanical hypersensitivity was established on week 6 of HSD feeding and maintained until at least 3 months. *n* = 20–22/group. **b** After 3 months of HSD feeding, the diet regime was replaced by a ND for an additional 2 months, but their HSD-induced decreased mechanical thresholds remained low, *n* = 16–18/group. **c** Mechanical hypersensitivity was not reversible even as their diet regime was shifted back to ND after 1.5 months of HSD feeding, *n* = 14–18/group. Data were combined from male and female mice. All data were presented as mean ± SEM. Data were analyzed by two-way ANOVA followed by Bonferroni post-tests, *p < 0.05, **p < 0.01, ***p < 0.001, ****p < 0.0001

To investigate the effect of an HSD on nociceptive pain behavior, we performed weekly von Frey tests on mouse hind paws to measure their sensitivity to mechanical stimuli. A decrease in the mechanical withdrawal thresholds of HSD-fed mice reached significance on week 6 and maintained this significance until the end of the experimental period (Fig. 2a). These results revealed that a long-term HSD could change individual’s pain sensitivity. We further observed that HSD-induced mechanical hypersensitivity is non-reversible, even when an HSD is shifted back to a ND. Mice who underwent 3 months of an HSD were put on a ND for an additional 2 months. Surprisingly, a ND was not able to restore the decreased paw withdrawal thresholds to normal levels (Fig. 2b). This was the case even with a 1.5-month HSD paradigm, wherein a ND was instituted immediately after the onset of mechanical hypersensitivity (Fig. 2c). This suggests that, once established, the mechanical allodynia triggered by HSD is long-lasting and cannot be reversed by a ND alone.

### An HSD triggers monocyte expansion and systemic inflammation

**Fig. 3:**
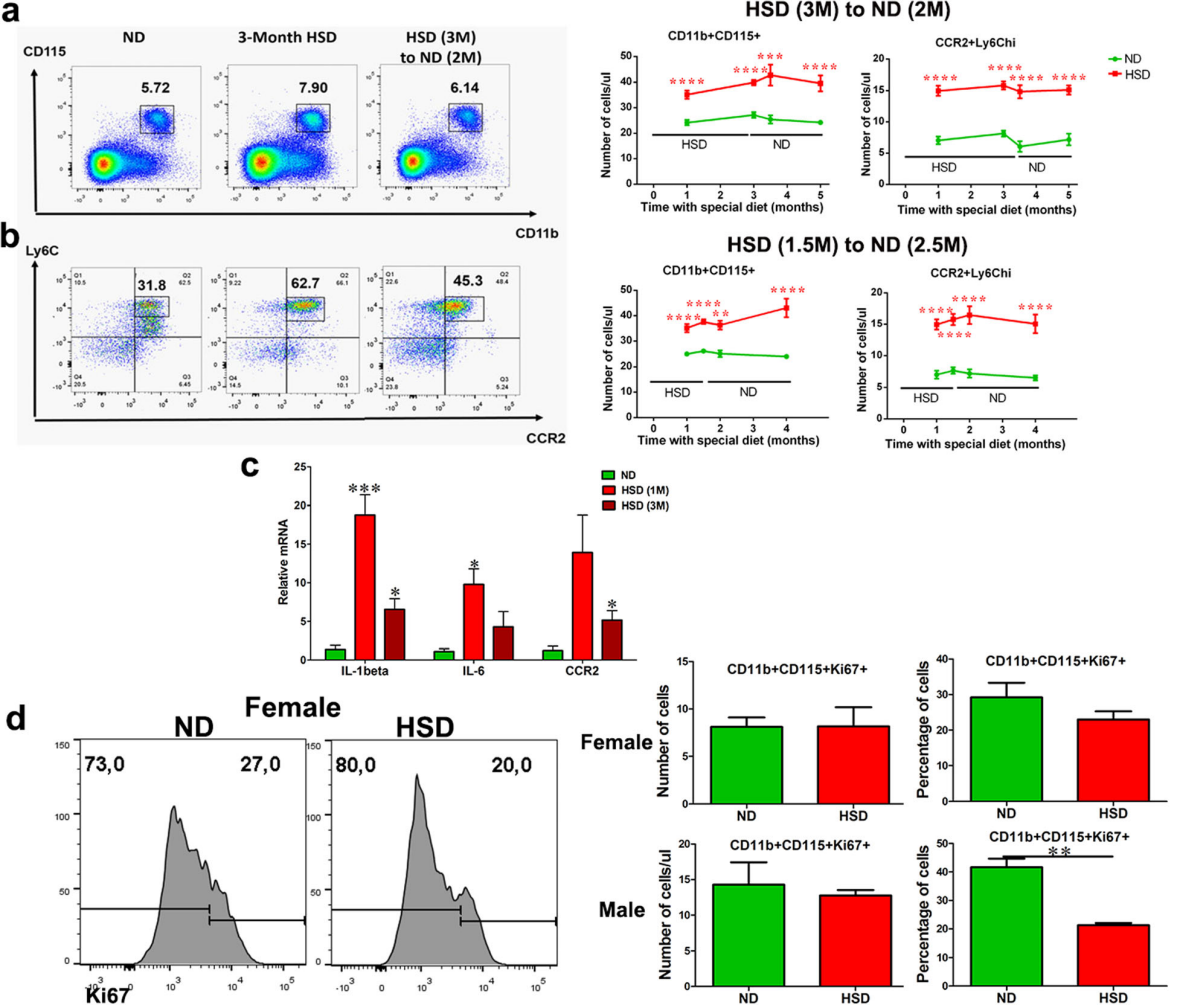
An HSD triggers monocyte expansion and systemic inflammation. **a** Flow cytometry dot plots of the frequency and the quantitative analysis of the absolute number showed a significant increase of CD11b^+^CD115^+^ monocytes in the blood of HSD mice. Such increase remained elevated even after returning to a ND, *n* = 10–14/group. **b** Flow cytometry dot plots of the frequency and the quantitative analysis of the absolute number showed a significant increase of CCR2^+^Ly6C^hi^ inflammatory subset in the blood of HSD mice. Such increase remained elevated even after returning to a ND, *n* = 10–14/group. **c** The mRNA expression of pro-inflammatory molecules, IL-1β, IL-6, and CCR2 at 1 month and 3 months of HSD feeding was determined by qPCR, *n* = 6–8/group. **d** A representative flow cytometry histogram showed the percentage of Ki67^+^CD11b^+^CD115^+^ monocytes in the blood of HSD/ND-fed mice. A quantitative analysis of absolute cell counts showed that HSD did not induce significant increases of cell proliferation, while due to the increase of total monocyte number, the percentage of Ki67^+^ monocytes was lower in the HSD group, *n* = 3–4/group. All data was presented as mean ± SEM, two-way ANOVA followed by the Bonferroni post-tests for **a**, **b**, un-paired *t* test for **c**, **d**, *p < 0.05, **p < 0.01, ***p < 0.001, ****p < 0.0001

It has been well-established that activated nerve macrophages and spinal microglia [20–22] contribute to chronic pain. In addition, high levels of salt can induce a specific activation of macrophages [8]. Therefore, we decided to scrutinize functional changes of myeloid cells under a long-term HSD to elucidate the underlying cellular mechanism of HSD-induced hypersensitivity. Indeed, an HSD induced a remarkable expansion of monocytes (monocytosis) in the blood. The absolute number of CD11b^+^CD115^+^ monocytes significantly increased in HSD mice (Fig. 3a) and there was an enhanced polarization toward CCR2^+^Ly6C^hi^ inflammatory monocytes (Fig. 3b) wherein the absolute number increased (Fig. 3a, b). Monocyte expansion was observed at 1 month of HSD feeding (Fig. 3a, b), which was before the onset of mechanical hypersensitivity. The number of circulating monocytes and pro-inflammatory subsets remained elevated over the course of HSD feeding (Fig. 3a, b). In addition, mice in the HSD group showed an elevated mRNA expression of pro-inflammatory cytokines interleukin I-β (IL-1β), interleukin-6 (IL-6), and the chemokine receptor CCR2 in the blood, which were more pronounced at 1-month of HSD feeding (Fig. 3c). To decipher the origin of the monocyte expansion, we assessed the number of proliferating monocytes in ND and HSD mice. Interestingly, the absolute number of Ki67^+^CD11b^+^CD115^+^ remained similar in both ND and HSD groups, in male and female mice. The percentage of proliferating monocytes was even lower in HSD mice as the total monocyte number increased (Fig. 3d). It is also worth noting that, on a ND, the monocyte proliferation rate in male mice (45.18 ± 4.305%) was higher than that in female mice (29.20 ± 4.133%) (Fig. 3d). Altogether, it suggests that the increase of circulating monocytes is not the result of cell proliferation; instead, it is mainly derived from enhanced egress from bone marrow. To understand why a ND cannot reverse HSD-induced hypersensitivity, we analyzed blood samples before and after such dietary shift. Irrespective of the length of HSD feeding (1.5 months or 3 months), 2 to 2.5 months of a ND was insufficient to reduce the number of circulating monocytes or normalize the inflammatory subset (Fig. 3a, b).

### An HSD activates peripheral nerve macrophages and spinal microglia

**Fig. 4:**
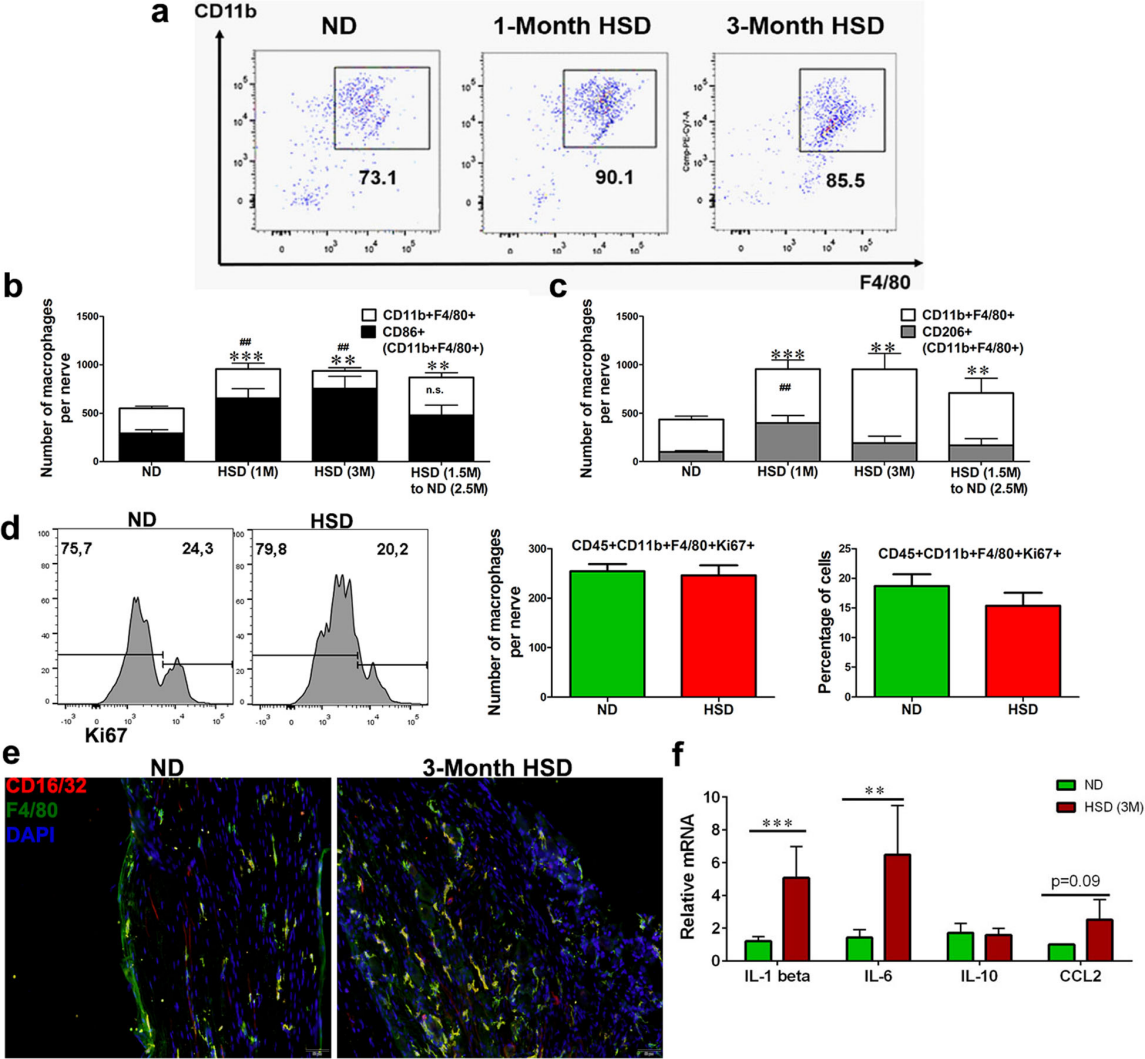
An HSD activates peripheral nerve macrophages. **a** Representative flow cytometry dot plots showed an increase in the frequency of peripheral nerve macrophages (CD45^+^CD11b^+^F4/80^+^) at 1 and 3 months of HSD feeding. **b** Histograms depicted the absolute macrophage (CD45^+^CD11b^+^F4/80^+^) number, as well as those that were CD86^+^ macrophages. A quantitative analysis indicated a significant increase at 1 and 3 months of HSD feeding, and the number remained elevated after shifting HSD-fed mice to a ND, *n* = 6–9/group. **c** Histograms depicted the absolute macrophage (CD45^+^CD11b^+^F4/80^+^) number as well as those that were CD206^+^ macrophages. An HSD favored the differentiation of CD86^+^ macrophages in the nerve more than those of CD206^+^ macrophages, *n* = 6–9/group. **d** A representative flow cytometry histogram showed the percentage of Ki67^+^macrophages (CD45^+^CD11b^+^F4/80^+^) in the sciatic nerves of HSD and ND fed mice. A quantitative analysis showed that HSD did not trigger significant macrophage proliferation in nerves, *n* = 6–8/group. **e** Longitudinal sections of mouse sciatic nerves were stained with CD16/32 (FcγII/III receptor), F4/80, and DAPI, showing an increased F4/80^+^ macrophage density in HSD nerves primarily colocalized with CD16/32. **f** Real-time quantitative PCR showed an elevated expression of IL-1β and IL-6 in the peripheral nerves at three months of HSD feeding, *n* = 6–10/group. Data were combined with from male and female mice. All data was presented as mean ± SEM and analyzed with an unpaired *t* test, **p < 0.01, ***p < 0.001; red and green ## represents statistics for CD86^+^ or CD206^+^ macrophages, respectively

To uncover the missing linkage among HSD-induced monocyte expansion, systemic inflammation, and mechanical hypersensitivity, we next explored the effect of an HSD on macrophages in peripheral nerves. Gated on CD45^+^ leukocytes, nerve macrophages were selected based on the cell surface markers CD11b and F4/80. Mice on an HSD showed a significant increase in the frequency and the number of CD11b^+^F4/80^+^ macrophages in the sciatic nerves (Fig. 4a, b). An HSD highly favored the development of CD86^+^ M1-like macrophages than that of CD206^+^ M2-like macrophages (Fig. 4b, c). A return to a ND was unable to bring the number of macrophages to the normal level; however, there was a trend toward a decrease in the number of CD86^+^ macrophages after the dietary change (Fig. 4b, c). While the total number of nerve macrophages increased after 1 month and 3 months of HSD feeding, the absolute number of Ki67^+^ proliferating macrophages remained similar in the sciatic nerves of ND and HSD mice (Fig. 4d), indicating the increase was mainly derived from recruitment. As a confirmation of the flow cytometry analysis, we stained the sciatic nerves with F4/80 to label nerve macrophages and CD16/32 (FcγII/III receptor) to highlight their activation status. As depicted in Fig. 4e, the density of F4/80^+^ macrophages in HSD mice was higher than that in ND mice. Most of these macrophages co-expressed CD16/32. These activated pro-inflammatory macrophages may contribute to the elevated expression of the pro-inflammatory cytokines IL-1β and IL-6 as seen in the peripheral nerves (Fig. 4f).

**Fig. 5:**
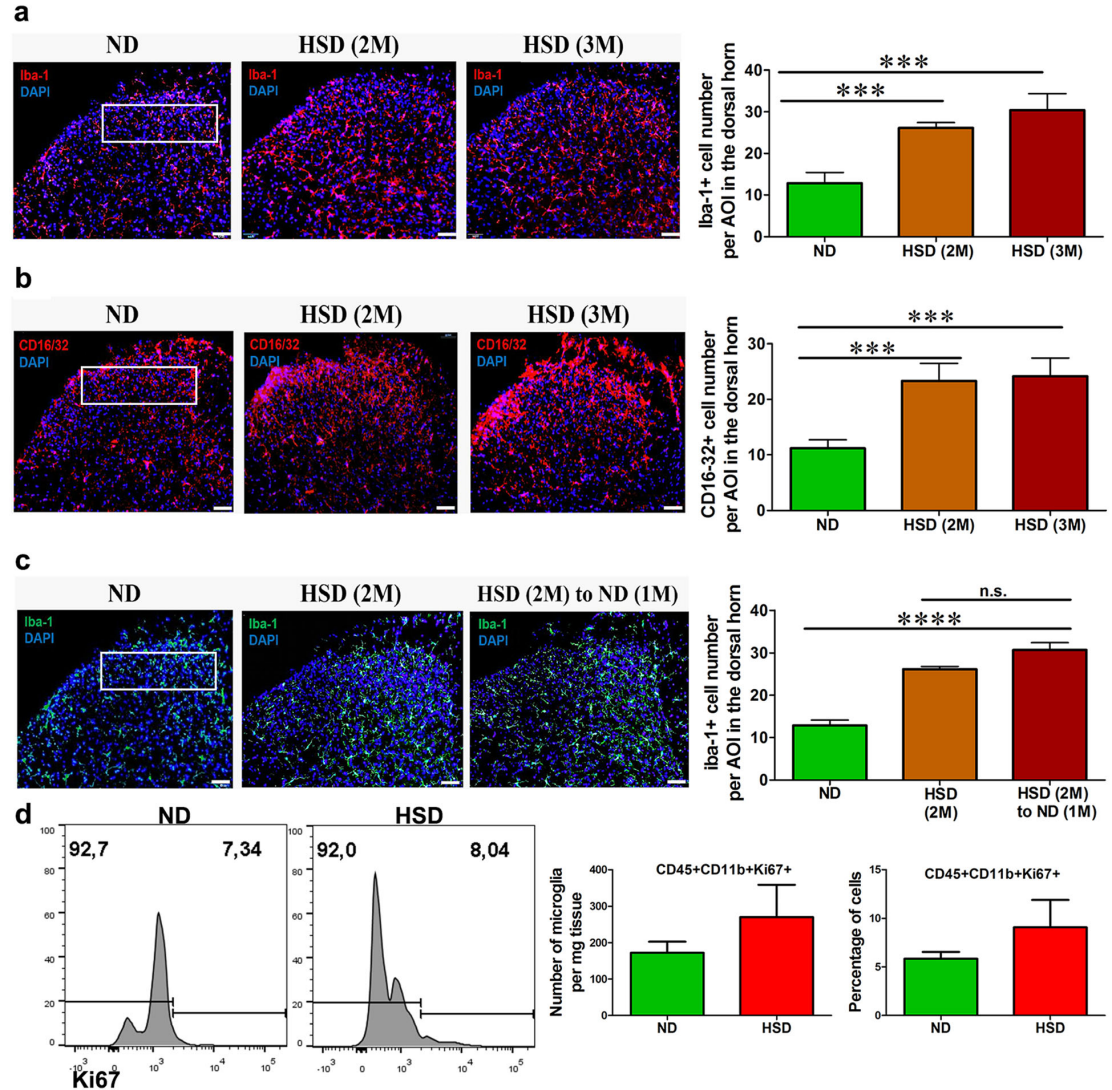
An HSD activates spinal microglia. **a** HSD feeding resulted in a significant increase in the number of Iba-1^+^ microglia in the lumbar spinal cord dorsal horn. **b** The expression of CD16/32 (FcγII/III receptor), an activation marker, was increased in the lumbar spinal cord of HSD fed mice. **c** Increased microglia density, as induced by HSD feeding, was maintained after returning the diet back to normal laboratory chow, *n* = 6–8/group. Scale bars: 50 μm. The outline indicates the area of interest (lamina I-III)) where microglia number was quantified. **d** A representative flow cytometry histogram showed the percentage of Ki67^+^ CD45^+^CD11b^+^ microglia in the lumbar spinal cord of HSD and ND mice. A quantitative analysis showed that an HSD did not trigger significant microglia proliferation, *n* = 6/group. Data were combined from male and female mice. All data was presented as mean ± SEM and analyzed with an unpaired *t* test, ***p < 0.001, ****p < 0.0001

We further investigated the effects of an HSD on spinal microglia, myeloid cells in the central nervous system. After 2 months of HSD feeding, there was a significant increase in the number of ionized calcium binding adaptor molecule 1 (Iba1)^+^ microglia in the dorsal horn (Fig. 5a), which was maintained for up to 3 months of HSD feeding. An HSD also induced a significant increase in CD16/32^+^ expression in the spinal cord (Fig. 5b). Similarly, to what has been observed in the circulating monocytes and nerve macrophages, HSD-induced spinal microglia activation persisted even after shifting the diet back to a ND (Fig. 5c). As was seen in the blood and sciatic nerves, an HSD did not significantly induce microglial cell proliferation in the spinal cord (Fig. 5d).

### Impeding CCR2 signaling successfully blocks myeloid cell trafficking and completely reverses HSD-induced mechanical allodynia

**Fig. 6:**
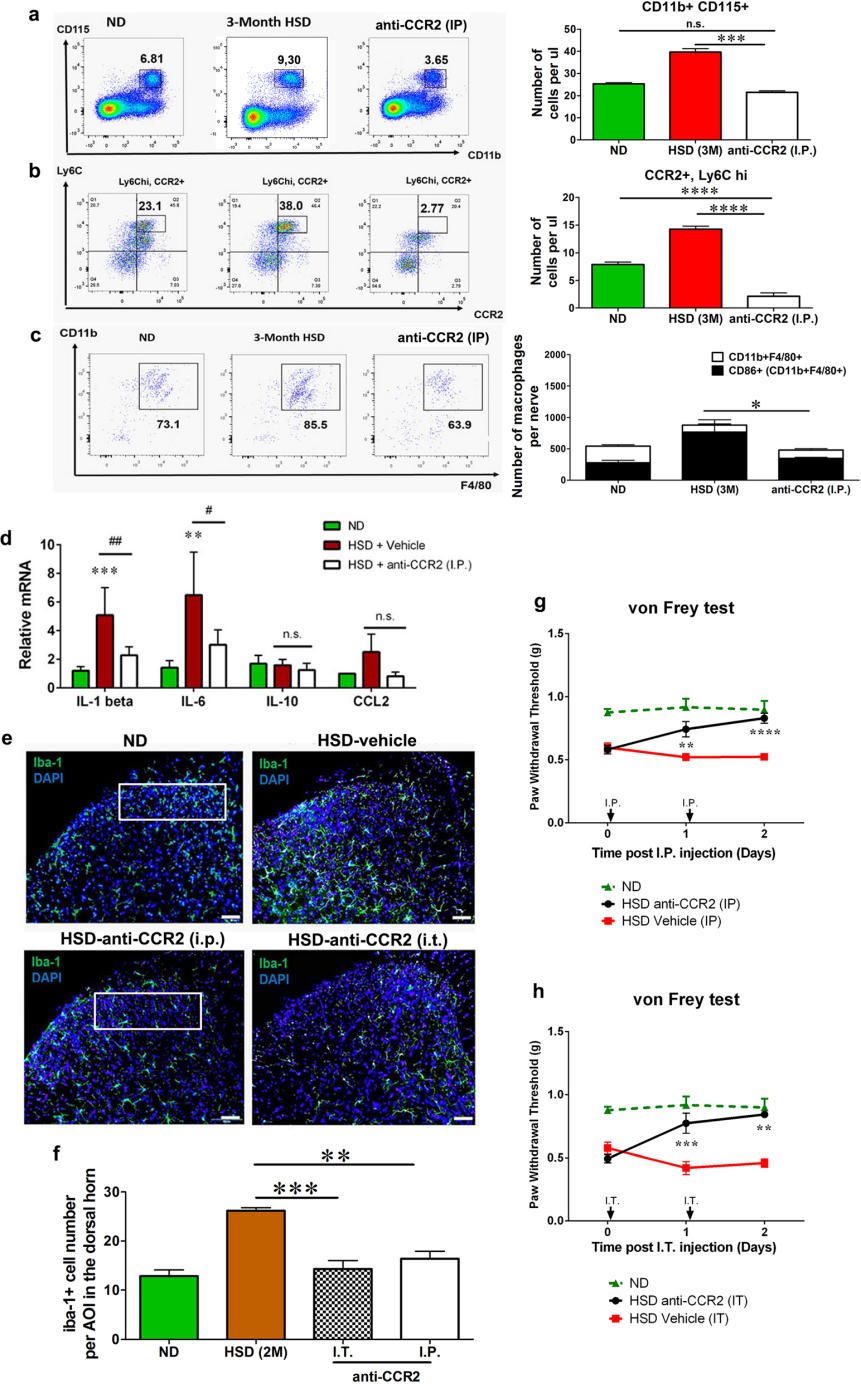
A CCR2 antagonist completely reversed the effects of an HSD on myeloid cell activation and mechanical hypersensitivity. **a A** CCR2 antagonist (RS 102895) was administered intraperitoneally for two consecutive days to mice after 3 months of HSD feeding. An anti-CCR2 (I.P.) treatment reduced the number of CD11b^+^CD115^+^ monocytes in the circulation to a normal level. **b** The anti-CCR2 (i.p.) treatment abolished the CCR2^+^Ly6C^hi^ subset in the blood. **c** The CCR2 antagonist reduced the number of CD45^+^CD11b^+^F4/80^+^ macrophages, as well as the number of CD86^+^ macrophages, in the peripheral nerve to a level comparable to the macrophages of ND-fed mice, *n* = 6–8/group. **d** The expression of IL-1β and IL-6 in the peripheral nerves was significantly reduced by I.P. anti-CCR2 treatment, while the expression of IL-10 and CCL2 was not affected, *n* = 6/group. **e**, **f** Both the intraperitoneal (I.P.) and intrathecal (I.T.) administrations of the CCR2 antagonist inhibited HSD-induced spinal microglia activation, *n* = 6/group. Scale bar: 50 μm. The outline indicates the area of interest (lamina I–III) where microglia number was quantified. **g**, **h** Two days of treatment with either the I.P. or I.T. administration of the CCR2 antagonist successfully rescued the HSD-induced decreased mechanical thresholds to a normal level, *n* = 9–11/group. Data with ND-fed mice was included (green dotted line) as reference. No significant difference was observed between male and female mice; therefore, the data was combined. All data was presented as mean ± SEM and analyzed with an unpaired *t* test for **a**–**d** and **f** or a two-way ANOVA followed by Bonferroni post-tests for **g** and **f**. *p < 0.05, **p < 0.01, ***p < 0.001, ****p < 0.0001

It is well known that CCR2 signaling is critical for myeloid cell trafficking [23]. The fact that an HSD significantly increased the number of CCR2^+^ monocytes in the circulation led us to speculate the importance of CCR2-mediated monocyte recruitment to the nervous system in altering mechanical sensitivity. RS102895, an antagonist of the β-subclass chemokine receptor CCR2 (15 mg/kg), was administrated intraperitoneally to HSD-fed male and female mice, once per day, for two consecutive days. Flow cytometry analysis revealed that the CCR2 antagonist successfully reduced the number of circulating monocytes to a normal level (Fig. 6a). There was a sharp decrease in the number of CCR2^+^Ly6C^hi^ inflammatory monocytes (Fig. 6b). Consistent with the effect in the circulation, a marked decrease in the frequency and number of CD11b^+^F4/80^+^ macrophages were observed in the sciatic nerves of mice treated with RS10289 along with a reduction of CD86^+^ macrophages (Fig. 6c). RS10289 treatment also downregulated mRNA expression of pro-inflammatory cytokines IL-1β and IL-6 in the blood (Fig. 6d). Worth pointing out, the number of spinal microglia was also reduced by i.p. RS10289 (Fig. 6e, f). Altogether, the systemic CCR2 antagonist effectively blocked the egress of monocytes from the bone marrow as well as the circulating monocyte trafficking to the nervous system. HSD-induced mechanical hypersensitivity was completely reversed following two consecutive administrations of RS10289 (Fig. 6g). To further ascertain the role of spinal microglia in HSD-induced mechanical hypersensitivity, we delivered RS102895 intrathecally in both male and female HSD mice. A 2-day treatment of RS102895 successfully reduced the number of spinal microglia (Fig. 6e, f) and reversed HSD-induced mechanical allodynia in male and female mice, without significant difference between sex (data from male and female mice were combined) (Fig. 6h). This indicates that blocking CCR2-mediated myeloid cell trafficking to the central nervous system is sufficient to abolish HSD-induced mechanical allodynia.

**Fig. 7:**
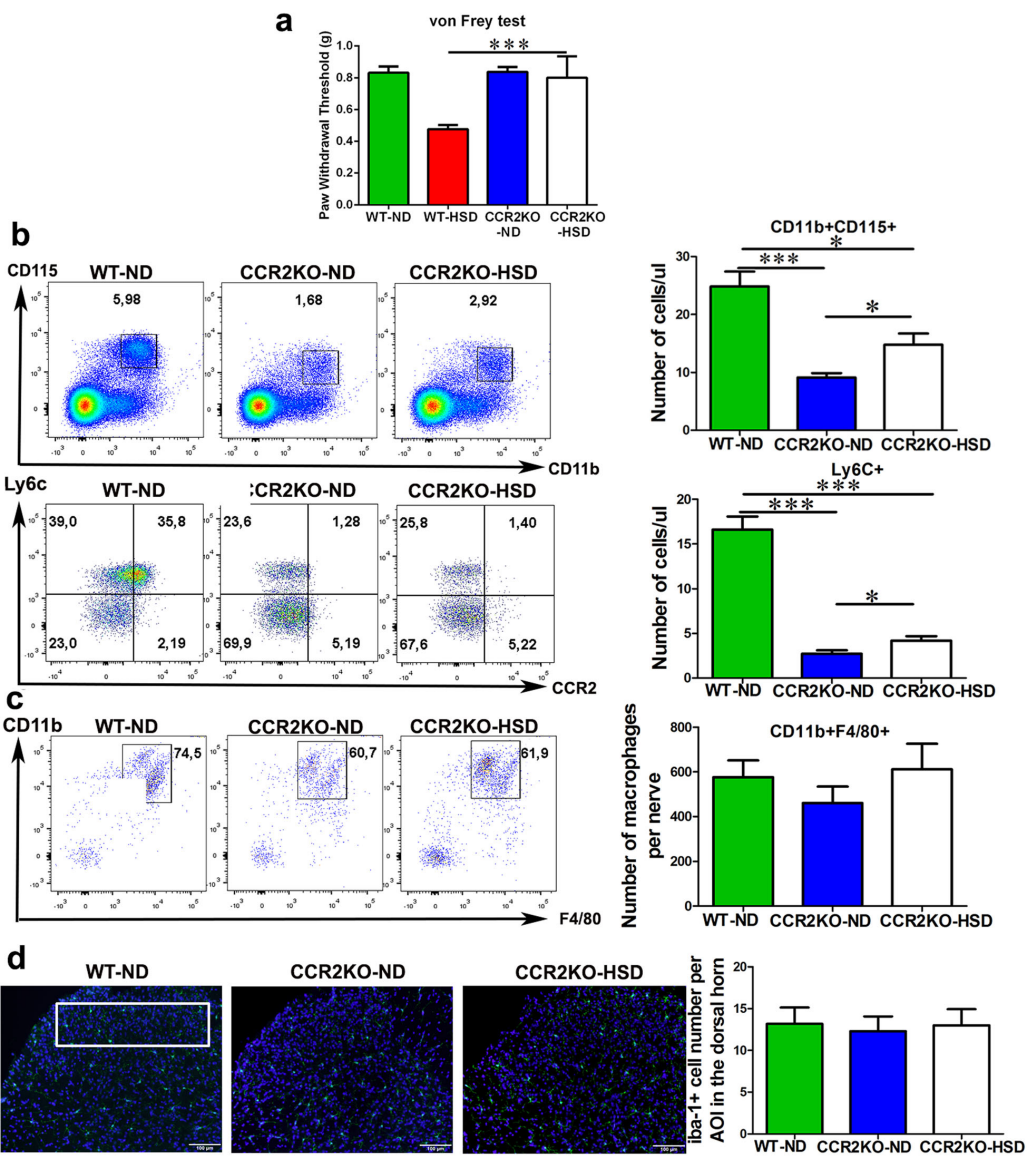
An HSD failed in inducing myeloid cell activation and enhancing pain sensitivity in CCR2 KO mice. **a** WT and CCRKO mice on a ND have similar mechanical thresholds. While 2 months HSD reduced paw withdrawal thresholds to mechanical stimuli in WT mice, it failed to do so in CCR2KO mice, *n* = 6–11/group. **b** When compared to WT mice, CCR2KO mice had significantly lower numbers of circulating monocytes, and only a slight increase was found when challenged with an HSD. The Ly6C^hi^ proinflammatory subset was significantly reduced in CCR2 KO mice and there was a slight increase in HSD mice. *N* = 6–11/group. **c** An HSD was not able to significantly increase the number of CD11b^+^F4/80^+^ macrophages in the peripheral nerves of CCR2KO mice. *N* = 6–11/group. **d** An HSD was not able to increase the number of Iba-1^+^ microglia in the lumbar spinal cord of CCR2KO mice. *N* = 6/group. Scale bar: 50 μm. The outline indicates the area of interest (lamina I–III) where microglia number was quantified. No significant difference was observed between male and female mice; therefore, the data was combined. All data was presented as mean ± SEM and analyzed with an unpaired *t* test. *p < 0.05, ***p < 0.001

**Fig. 8:**
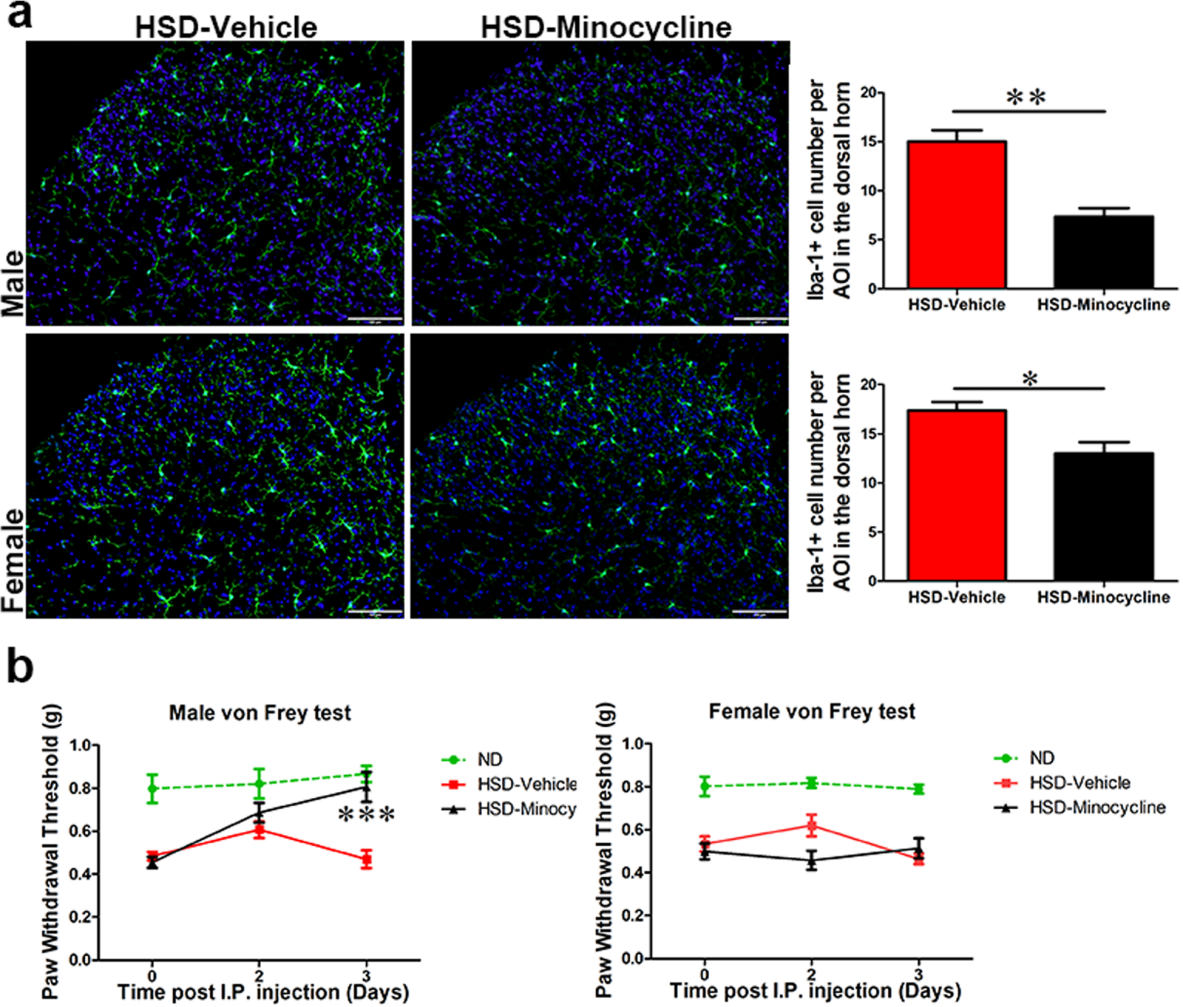
Spinal microglia is not involved in HSD-induced mechanical hypersensitivity in female mice. **a** Minocycline significantly reduced the number of Iba-1^+^ microglia in the spinal cord dorsal horns in both male and female mice, *n* = 3/group. Scale bar: 100 μm. The outline indicates the area of interest (lamina I–III) where microglia number was quantified. **b** Minocycline increased the paw withdrawal thresholds in male HSD mice, but not in female HSD mice. Data with ND-fed mice was included (green dotted line) as reference. *N* = 6–8/group. All data was presented as mean ± SEM and analyzed with an unpaired *t* test for **a** or a two-way ANOVA followed by Bonferroni post-tests for **b**. **p < 0.01, ***p < 0.001

Furthermore, we placed male and female CCR2 KO mice on an HSD or a ND for 2 months. Wild-type and CCR2 KO mice on ND exhibited similar mechanical sensitivity (Fig. 7a). An HSD failed to enhance mechanical sensitivity in CCR2 KO mice (Fig. 7a). When compared to wild-type mice on a ND, a deficiency of CCR2 resulted in a significantly lower number of circulating monocytes, which was still slightly increased after 2 months of HSD feeding (Fig. 7b). The proinflammatory subset (Ly6C^hi^) was also greatly reduced in CCR2 KO mice (Fig. 8b). An HSD failed in inducing an increase of nerve macrophages nor spinal microglia in CCR2 KO mice (Fig. 7c, d). No significant sex difference was observed in term of cellular response (monocytes, nerve macrophages, spinal microglia), nor in pain behavioral changes in male and female CCR2KO HSD mice. The findings depicted here were combined data from male and female mice.

### Spinal microglia in female mice do not contribute to HSD-induced mechanical hypersensitivity

It appears that in both male and female mice, blocking CCR2 signaling inhibited HSD-induced spinal microglia activation and reversed mechanical hypersensitivity. However, CCR2 is reported to be expressed not only on microglia but also on other types of cell, including sensory neurons in dorsal root ganglia [24]. To further confirm the contribution of spinal microglia in HSD-induced hypersensitivity, we treated HSD male and female mice with minocycline (I.P., 25 mg/kg, b.w., daily for 3 days). While in both male and female mice, the number of Iba-1^+^ microglia was significantly less abundant in minocycline-treated group (Fig. 8a); minocycline only increased paw withdrawal thresholds in male, but not female mice (Fig. 8b). Thus, spinal microglia is not required for HSD-induced mechanical hypersensitivity in female mice.

## Discussion

While the human body cannot live without salt (sodium), many people consume too much without realizing the consequences. The current study demonstrates that the long-term over-consumption of salt has a direct impact on basic physiological functions. An HSD confers mice to an inflamed status that includes monocytosis in the circulation, an activation of macrophages and microglia in the nervous system. Mice on an HSD are more sensitive to mechanical stimuli than those on a ND. Once established, HSD-induced myeloid cell activation and decreased mechanical thresholds are long-lasting and will be maintained even after the HSD is replaced by a ND. Long-term HSD-associated hypersensitivity is driven by a CCR2-dependent mechanism. Impeding CCL2/CCR2 signaling prevents monocyte egress from bone marrow as well as myeloid cell recruitment to the nervous system. HSD fails in inducing mechanical hypersensitivity in CCR2-deficient mice. A CCR2 antagonist abolishes HSD-triggered painful response in wild-type mice. However, spinal microglia is only required for HSD-associated mechanical hypersensitivity in male but not in female mice.

Salt is recognized as a risk factor for high blood pressure, heart failure, and kidney diseases. Beyond these notable effects, an increasing amount of evidence demonstrates that excessive salt affects the immune system. In healthy humans, an increase in dietary salt intake for only few days, e.g., 7–50 days, is sufficient to induce an expansion of CD14^+^CD16^+^ monocytes, which can be reversed by a low-salt diet [25, 26]. Cytokine production was positively correlated with the amount of salt consumed and the dynamics of circulating monocytes [25, 26]. In line with the human data, our study revealed a remarkable HSD-triggered monocyte expansion, in particular, an increase of the CCR2^+^Ly6C^hi^ inflammatory subset accompanied by an increased cytokine level in the blood. However, when compared to human studies involving a few days of an increase in salt intake, the long-term HSD paradigm (1.5 to 3 months) used in the current mouse study resulted in irreversible consequences. Data from both human and animal studies suggests that monocytes in the blood could be directly affected by dietary salt intake.

Apart from the influence on circulating monocytes, high levels of salt also impact tissue macrophages. Zhang et al. [8] treated human monocytes-derived macrophages and murine bone marrow-derived macrophages with high concentrations of salt (51 mM). They defined a specific high salt-induced macrophage activation state [M(Na)] that is different from that induced by lipopolysaccharide (LPS) [M(LPS)] or interferon-γ (IFNγ) [M(IFNγ)]. Their data also suggested that high levels of salt promote the adhesion and recruitment of monocytes to target organs and boost proinflammatory cytokine/chemokine secretion from monocytes/macrophages [8]. In addition, Binger et al. [14] reported that increased salt intake inhibits alternative macrophage differentiation from the M2-like functional phenotype. We observed that similar to monocytosis, the number of macrophages in the peripheral nerves and microglia in the spinal cords of HSD-fed mice significantly increased. The majority of these macrophages/microglia exhibited an inflammatory phenotype. Although unaddressed in the current study, it has been reported that high salt levels also imbalance adaptive immunity by inducing pathologic Th17 cells [27] and promoting a proinflammatory Treg phenotype with heightened IFNγ secretion and a diminished suppressive capacity [28]. While a balanced immune response requires precise regulation, the data from this study and from previous literature indicated that salt may tilt the balance toward a proinflammatory phenotype. An HSD could exacerbate inflammation and autoimmune diseases using a double punch: an increase in inflammatory functions and an inhibition of regulatory function in both myeloid cells and T lymphocytes cells [29].

An HSD did not increase the number of Ki67^+^ monocytes, macrophages, or microglia, which suggests that an HSD does not trigger significant myeloid cell proliferation and instead initiates cell migration. This hypothesis was confirmed by the fact that CCR2 antagonist completely reversed the number of circulating monocytes in HSD mice to a normal level, abolished the CCR2^+^Ly6C^hi^ subset, and normalized the number of nerve macrophages and spinal microglia. An HSD failed to induce myeloid cell expansion in CCR2 KO mice. The chemokine receptor CCR2 is highly expressed by a majority of myeloid cells in physiological conditions. It has a crucial role in monocyte homeostasis because it controls monocyte egress from bone marrow under both resting and infection/inflammation conditions [23, 30]. A blockade of CCR2 signaling leads to a reduction in circulating monocytes and a concomitant sequestration of bone marrow monocytes and monocyte precursors [30]. CCR2 is also necessary for efficient monocyte recruitment from the blood to inflamed tissue [31, 32]. CCR2 is required for macrophage infiltration in nerve degeneration and regeneration process [33], and for nerve injury-triggered spinal microglia activation [34, 35]. Altogether, our study concluded that long-term high-salt intake drives monocyte mobilization from bone marrow to the blood stream in a CCR2-dependent manner. When CCR2 signaling is inhibited, the reduced recruitment of monocytes to nervous tissues may be attributed to the reduced number of circulating monocytes and the reduced ability of monocytes to move from the blood to the tissues.

It has been well-recognized that there exists a dynamic crosstalk between the immune system and the nervous system. Inflammation mediated by innate immune cells, primarily monocyte-derived macrophages and microglia in the central nervous system, contributes to the development and maintenance of chronic pain [6]. The evidence in the current study clearly demonstrated that mice who maintain an inflammatory status due to the long-term over-consumption of salt have altered pain thresholds. They feel more pain than those on a ND. Furthermore, the study uncovered a CCR2-dependent underlying mechanism. While blocking the CCR2 signaling has successfully arrested the development of HSD-triggered monocytosis and impeded nerve macrophages/spinal microglia activation, mechanical thresholds were coincidently normalized, in both male and female mice. Myeloid cells in CNS disease and injury are essentially composed of infiltrating monocyte-derived macrophages (MDMs) and resident microglia; both types of cells are thought to be critical to functional outcomes. Some recent studies revealed that MDMs and microglia directly communicate with one another and differentially modulate each other’s functions [36]. For example, infected peripherally derived macrophages can prime naive microglial cells causing microglia to express a range of inflammatory mediators [37, 38]. In the case of spinal cord injury, the arrival of monocyte-derived macrophages coincides with the downregulation of microglial inflammation and phagocytic functions [39]. Although HSD does not trigger significant resident microglia proliferation, it remains to be determined whether and how HSD-induced monocyte-derived macrophages talk to resident microglia and contribute to their activation. It is worth noting that while inhibiting spinal microglia activation by minocycline completely rescued mechanical thresholds to normal level in male mice, it failed in female mice, indicating that spinal microglia in female mice are not required in HSD-induced hypersensitivity. The observed sex difference is in line with microglia contribution in other chronic pain condition [40]. Our results thus reveal that, in HSD-associated hypersensitivity, CCR2 contribution is not exclusively mediated by myeloid cells. The fact that pain thresholds can be normalized by blocking CCR2 signaling, but not by inhibiting spinal microglia activation in female mice suggests that the effect of CCL2/CCR2 could be mediated by other types of cells, e.g., sensory neurons. Although the expression of CCR2 is very low in naive sensory neurons, an upregulation has been reported in some neuropathic condition [24, 41]. The direct evidence of neuronal CCR2 expression and contribution in HSD condition is worth for future investigation.

It is intriguing that HSD-induced hypersensitivity and myeloid cells alterations were not reversible even the HSD was replaced by a ND. Our data suggests that sustained hypersensitivity could be the consequence of the long-lasting increase of CCR2^+^ myeloid cells. We speculate that the latter could be maintained by systemic inflammation following long-term over-consumption of salt. The duration of HSD should be an important factor, since two human studies involving a few days of an increase in salt intake (in a ~ 70-year life span) showed that HSD-induced monocytosis and inflammation are indeed reversed by low-salt diet [25, 26], although they did not assess individuals’ pain sensitivity. The paradigm used in the current mouse study, 1.5 to 3 months HSD feeding (in a 2-year life span), is significantly much longer, reflecting changes in lifestyle, instead of occasional, temporary too much salt in the diet.

## Conclusion

Overall, this study issued an important message that, in addition to genetic determinants, environmental factors, such as diet, may also alter an individual’s pain sensitivity. Long-term over-consumption of salt may decrease a subject’s pain thresholds causing them to feel more pain than others. Chemokine receptor CCR2-mediated myeloid cell trafficking and the associated inflammation are important players in the cascade.

## Data Availability

The datasets during and/or analyzed during the current study available from the corresponding author on reasonable request.

## Abbreviations

HSD: High-salt diet
ND: Normal diet
CCR2KO: CCR2 knock out
PCR: Quantitative real-time polymerase chain reaction
IHC: Immunohistochemistry
AOI: Area of interest
I.P.: Intraperitoneally
I.T.: Intrathecally
LPS: Lipopolysaccharide
Treg: T regulatory
EAE: Experimental autoimmune encephalomyelitis
IL-1β: Interleukin I-β
IL-6: Interleukin-6
Iba-1: Ionized calcium binding adaptor molecule 1
b.w.: Body weight
IFNγ: Interferon-γ
